# Undiagnosed Rare Genetic Disorders: Importance of Functional Characterization of Variants

**DOI:** 10.3390/genes14071469

**Published:** 2023-07-19

**Authors:** Muhammad Umair, Ahmed Waqas

**Affiliations:** 1 Medical Genomics Research Department, King Abdullah International Medical Research Center (KAIMRC), King Saud Bin Abdulaziz University for Health Sciences, Riyadh 11426, Saudi Arabia; 2 Department of Zoology, Division of Science and Technology, University of Education, Lahore 54770, Punjab, Pakistan; ahmed.waqas@ue.edu.pk

Rare Genetic Disorders (RGDs) are defined as disorders that affect less than 1 in 2000 people, and collectively affect more than 300 million people worldwide [1]. The Online Mendelian Inheritance in Man (OMIM) database reports over 10,000 RGDs, while Orphanet reports 6172 unique RGDs, 70% of which have juvenile onset [1,2]. RGDs are heterogeneous in nature and can be managed through a variety of approaches, ranging from molecular biology to epidemiology, and include clinical assessment, biostatistical analysis, and laboratory experiments.

Establishing a diagnosis is one of the many difficulties encountered by RGD patients and their families. Patients frequently remain undiagnosed for years due to the rarity of their conditions, the sheer number of disease entities, and the varied manifestations of their symptoms. As a result, the burden of undetected RGDs has increased globally. For a patient and their family, obtaining the right diagnosis can have major ramifications, as it can shed light on the etiology, course, and prognosis of the disease, and it can make it easier for affected families to connect with support networks and seek genetic counseling. Additionally, it can advance knowledge of disease pathophysiology, and thus, enhance therapeutic approaches [3].

In the past decade, next-generation sequencing (NGS) technologies such as whole-exome sequencing (WES) and whole-genome sequencing (WGS) have enabled the identification of disease-causing variants for a large number of unsolved RGDs. The diagnostic accuracy of NGS-based sequencing varies from 25 to 65%, depending on the technology employed, the disease group studied, the clinical indications, the patients’ age groups and family structures, and the variants of filtration methods used [4]. Even though NGS-based sequencing offers a better opportunity to elucidate the genetic causes of RGDs, a proportion of individuals remain undiagnosed. Therefore, within this Special Issue, we compile a collection of articles that present emerging approaches to facilitating genetic diagnoses and that discuss the impacts of identifying the genetic and molecular causes of RGDs. The main objective of this Special Issue is to gather information associated with disease-causing variants and consider their associations with different RGDs. The articles included in herein offer interesting insights into the range of biological processes involved in different RGDs.

The research topic was established in April 2022, and we accepted manuscripts until July 2023. In the first paper included in the present Special Issue, Rehman et al. [5] reported on a consanguineous Pakistani family who had an autosomal recessive stuttering disorder with a neurodevelopmental disorder inherited in autosomal recessive manner. Using WES, they revealed a biallelic splice site variant (c.916+1G>A) in the ARMC3 (Armadillo Repeat-Containing 3) gene (NM_173081.5). They further revealed that the splice site variant might cause exon 8 to be skipped and result in the loss-of-function (L.O.F) variant, which might undergo NMD (nonsense-mediated decay). In the second study, Zaman et al. [6], reported on two families with Hermansky–Pudlak syndrome (HSP). WES analysis revealed a novel biallelic nonsense variant (c.2766T>G) in *HSP3* (NM_032383.4) in family ALB-09 and a biallelic 5bp deletion (c.1180_1184delGTTCC) variant in the HSP4 gene (NM_001349900.2) in family ALB-10. Both of these variants might result in L.O.F and NMD. In the third study, Hovland et al. [7] conducted functional analyses of rare BRCA1 missense variants that are located within and outside protein domains. They revealed that the variants located outside the BRCT, RING, and coiled–coiled domains could affect BRCA1 protein function. Similarly, Umair et al., 2023, reported on a novel biallelic missense variant (c.155T>A; p.Phe52Tyr) in the SLCO2A1 gene that caused digital nail clubbing in a single affected individual. Sanger segregation and 3D protein modeling revealed the pathogenic nature of the identified variant.

In the fifth study, Bakar et al. [8] reported on a family with Dyggve–Melchior–Clausen Syndrome, which results from disease-causing variants in the DYM gene. Homozygosity mapping, linkage analysis, and Sanger sequencing of the DYM gene revealed a novel biallelic disease-causing variant (c.1205T>A; p.Leu402Ter) in the DYM gene. Furthermore, Nawaz et al. [9] reported on ten families with classical Bardet–Biedl syndrome (BBS). Using WES, they revealed six previously reported and four novel mutations in 10 different families. Their study extended the genetic and phenotypic spectrum of BBS and supported data suggesting that variants in these genes are associated with the development of multi-systemic human genetic disorders. Similarly, Umair et al. [10] reported a proband with the hallmark features of isolated digital clubbing. WES data analysis revealed a homozygous missense variant (c.155T>A; p.Phe52Tyr) in the *SLCO2A1* gene. Their study shed light on the pathogenesis of mutated *SLCO2A1* in digital clubbing that may lead to exciting developments in our understanding of this gene’s role in nail development/morphogenesis [10].

In recent years, variant detection technologies have evolved dramatically, and large-scale data associated RGDs have been generated. After a patient has received an initial disease confirmation from a clinical lab, followed by radiological, biochemical, and MRI examinations, they are referred for molecular testing. These tests include cytogenetic analysis; karyotyping for the identification of gross chromosomal aberrations; CGH microarray to identify duplications, deletions, aneuploidy, and loss of heterozygosity; and targeted gene panels and next-generation sequencing (WES/WGS) to identify point mutations, which are confirmed using standard Sanger sequencing [3].

An organism tolerates most of the genetic variants that we observe, but some variants can change the function of a gene’s product. Functional genetic variation studies aim to understand the molecular mechanisms and pathways that link genotype to phenotype. In silico tools can be used to check the pathogenicity of the identified variants, further confirm the identified abnormalities, and establish a molecular diagnosis. The pathogenic nature of the abnormality or variant is checked according to the ACMG guidelines. They classify variants and chromosomal abnormalities into five categories, i.e., pathogenic, likely pathogenic, variants of uncertain significance (VUS), likely benign, and benign. The pathogenicity of the VUS is further confirmed using functional studies, which are chosen depending upon the nature of the gene.

International collaboration and the performance of collaborative functional studies on novel candidate genes are also challenges. Different databases have been established that allow scientists to share their patients’ preliminary data and connect with other scientists working on the same gene/disorder. These include several famous databases, such as DECIPHER (https://www.ddduk.org; accessed on 9 July 2023), GeneMatcher (https://genematcher.org/; accessed on 9 July 2023), PhenomeCentral (https://phenotips.org; accessed on 9 July 2023), MyGene2 (https://mygene2.org/; accessed on 9 July 2023), PatientMatcher (https://www.ki.se; accessed on 9 July 2023), and seqr (https://seqr.broadinstitute.org; accessed on 9 July 2023). Some of the major concerns regarding online gene matching databases include the possibility of self-matches, whereby two independently submitted cases represent the same patient. Self-matches are common, as a patient with a particular RGD may have been evaluated at multiple research centers. Efforts to establish unique identifiers will certainly help rectify this problem. Similarly, functional studies require a lot of time for validation. Reporting on a novel candidate disease-causing gene often requires a substantial amount of time from the initial match to publication. While it is essential to gather a sufficient amount of supporting evidence for disease causality before publication, the length of time in which potential results are kept private prior to publication is increasing.

It is apparent that NGS diagnostic applications can help to achieve quick and accurate molecular diagnoses; however, a translational gap associated with the clinical implementation of NGS-based testing still exists, mainly due to a lack of technical scientists and/or a scarcity of infrastructure. Public health policies for RGD patients should be revised, and proper diagnoses and safe, affordable, FDA-approved medications provided. Furthermore, data sharing and international collaboration among clinicians and scientists are advancing our knowledge of genomic medicine, making rare Mendelian genetics and complex diseases exciting fields of study. Data sharing in a manner that protects patient privacy and expedites novel gene discovery is essential to the future gene-therapy of RGDs. Thus, the improvement of basic molecular diagnosis and investment in the gene therapy-based research might result in the prevention of prevalent RGDs.
